# Genetic Basis of Haloperidol Resistance in *Saccharomyces cerevisiae* Is Complex and Dose Dependent

**DOI:** 10.1371/journal.pgen.1004894

**Published:** 2014-12-18

**Authors:** Xin Wang, Leonid Kruglyak

**Affiliations:** 1 Department of Molecular Biology, Princeton University, Princeton, New Jersey, United States of America; 2 Lewis-Sigler Institute for Integrative Genomics, Princeton University, Princeton, New Jersey, United States of America; 3 Department of Human Genetics, University of California, Los Angeles, Los Angeles, California, United States of America; 4 Department of Biological Chemistry, University of California, Los Angeles, Los Angeles, California, United States of America; 5 Howard Hughes Medical Institute, Chevy Chase, Maryland, United States of America; The University of North Carolina at Chapel Hill, United States of America

## Abstract

The genetic basis of most heritable traits is complex. Inhibitory compounds and their effects in model organisms have been used in many studies to gain insights into the genetic architecture underlying quantitative traits. However, the differential effect of compound concentration has not been studied in detail. In this study, we used a large segregant panel from a cross between two genetically divergent yeast strains, BY4724 (a laboratory strain) and RM11_1a (a vineyard strain), to study the genetic basis of variation in response to different doses of a drug. Linkage analysis revealed that the genetic architecture of resistance to the small-molecule therapeutic drug haloperidol is highly dose-dependent. Some of the loci identified had effects only at low doses of haloperidol, while other loci had effects primarily at higher concentrations of the drug. We show that a major QTL affecting resistance across all concentrations of haloperidol is caused by polymorphisms in *SWH1*, a homologue of human oxysterol binding protein. We identify a complex set of interactions among the alleles of the genes *SWH1*, *MKT1*, and *IRA2* that are most pronounced at a haloperidol dose of 200 µM and are only observed when the remainder of the genome is of the RM background. Our results provide further insight into the genetic basis of drug resistance.

## Author Summary

Variation in response to a drug can be determined by many factors. In the model organism baker's yeast, many studies of chemical resistance traits have uncovered a complex genetic basis of such resistance. However, an in-depth study of how drug dose alters the effects of underlying genetic factors is lacking. Here, we employed linkage analysis to map the specific genetic loci underlying response to haloperidol, a small molecule therapeutic drug, using a large panel of segregants from a cross between two genetically divergent yeast strains BY (a laboratory strain) and RM (a vineyard strain). We found that loci associated with haloperidol resistance are dose-dependent. We also showed that variants in the oxysterol-binding-protein-like domain of the gene *SWH1* underlie the major locus detected at all doses of haloperidol. Genetic interactions among genes *SWH1*, *MKT1*, and *IRA2* in the RM background contribute to the differential response at high concentrations of haloperidol.

## Introduction

The budding yeast *Saccharomyces cerevisiae* has become a powerful model for elucidating fundamental principles and mechanisms of complex trait genetics [1]. Many quantitative trait loci (QTL) – and the causal genes underlying these loci – have been identified for diverse biological processes, including gene expression [2]–[4], high-temperature growth [5]–[8], DNA damage repair [9], sporulation efficiency [10]–[12], and drug sensitivity [7], [13], [14]. In studies of chemical resistance traits, compound concentrations with the highest heritability are typically selected for further analysis [15]. However, the extent to which the genetic architecture underlying the response to a drug is specific to the drug dose is a major open question.

Following initial observations of complex and dose-dependent inheritance patterns of the response to the small molecule haloperidol, we set out to investigate the genetic basis of haloperidol resistance as a function of dose. Haloperidol is a psychoactive drug that binds to dopamine and serotonin receptors in humans [16], and is widely used for treating schizophrenia. In *Saccharomyces cerevisiae* (which does not contain the pharmacologically relevant haloperidol targets), haloperidol exerts effects on vesicle transport and amino acid metabolism [17], demonstrating perturbations of fundamental cellular physiology upon exposure to the drug. Haloperidol, a cationic amphiphilic drug, has been shown at concentrations of 10–200 µM to cause defects in phospholipid metabolism/transport [18], [19] and trigger autophagy upon accumulation [20] in yeast, and to result in degradation of membranes [21] *in vitro*. Haloperidol was also found to inhibit both sterol Δ^8,7^ isomerase (Erg2) and C-14 reductase (Erg24) activities in yeast [22], [23]. An early biochemical study showed that haloperidol binds to Erg2 in yeast, and causes decreased ergosterol levels [23]. Erg2 functions in the ergosterol biosynthesis pathway, suggesting haloperidol's interference with sterol metabolism and trafficking.

Here, we used a large panel of 1008 segregants from a cross between a laboratory strain BY4724 (hereafter referred to as BY) and a vineyard strain RM11-1a (hereafter RM) to study yeast growth in haloperidol. We identified a total of nine genomic loci associated with resistance to haloperidol with different dose-specificity. We further identified *SWH1* as a major gene contributing to resistance to haloperidol at all concentrations, and showed that variants within its oxysterol binding protein (OBP)-like domain are responsible for resistance. We also showed that variants in *MKT1* and *IRA2* underlie loci that have effects predominantly at high haloperidol concentrations, and found complex, background-dependent genetic interactions among the allelic states of *SWH1*, *MKT1*, and *IRA2*. This study sheds light on the contribution of QTL-dosage interaction to chemical resistance in yeast, and the complexity of the underlying sources of variation in quantitative traits.

## Results

### Haloperidol induces pH dependent sensitivity and vacuole defects

**Figure 1:**
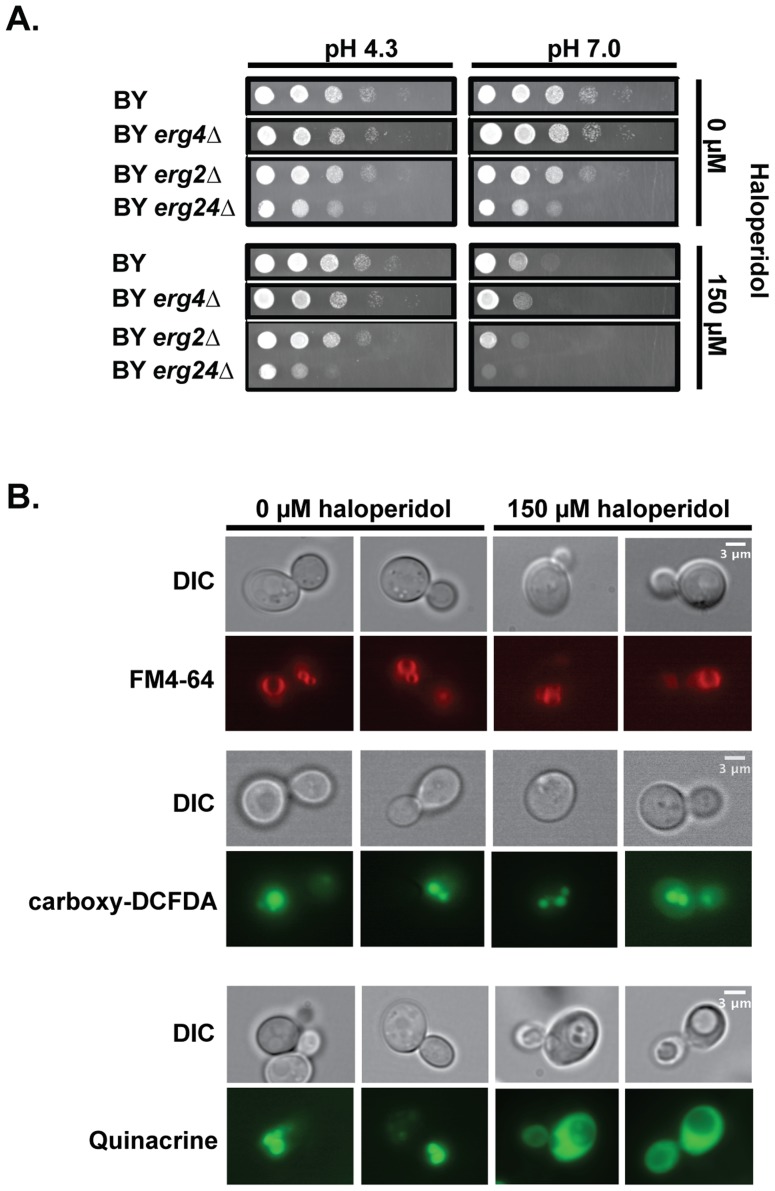
Haloperidol induces pH dependent sensitivity and other biological effects in yeast. (A) Growth at different pH values and haloperidol concentrations. *pH 4.3*: YPD at pH value 4.3; *pH 7.0*: YPD at pH value 7.0. Saturated cultures in liquid YPD were serially diluted 1∶10 before pinning onto agar plates. Plates were incubated at 30°C for 24–48 hr. (B) Haloperidol causes vacuole acidification related impairment. Treated versus untreated cells were stained (see *Methods*) with FM4-64 (stains vacuole membrane), carboxy-DCFDA (diffuses into vacuole) after 2 hours of haloperidol exposure, and quinacrine (accumulates in acidic compartments) after 6 hours of treatment with haloperidol at 150 µM.

To assess the biological effects of haloperidol in *S. cerevisiae*, we examined susceptibility of the laboratory strain BY carrying gene deletions *erg2Δ*, *erg24Δ*, or *erg4Δ* to haloperidol in rich medium (Fig. 1A). Erg2, Erg24, and Erg4 make up three important steps in the ergosterol biosynthesis pathway in yeast [24]. BY *erg2Δ* and BY *erg24Δ* strains had growth defects in rich medium, but neither was completely resistant to haloperidol. Erg4 catalyzes the final step in the ergosterol biosynthesis pathway, and it has been shown that *erg4Δ* mutants lack detectable levels of ergosterol [25]. Deleting *ERG4* did not eliminate the sensitivity to haloperidol (Fig. 1A); thus, haloperidol has biological effects other than those on the ergosterol pathway [17].

Similar to previous observations with other cationic amphiphilic drugs [18], [20], we found sensitivity to haloperidol to be pH dependent (Fig. 1A). Acidic pH ( = 4.3) [26] completely rescued growth in the presence of 150 µM haloperidol (Fig. 1A). pH related phenotypes are often indicative of vacuole-related defects [27]. Staining of the vacuole and vacuolar membrane of haloperidol-treated cells showed that vacuoles were intact (Fig. 1B). Measuring acidity with fluorescent dye quinacrine (which accumulates in acidic compartments) revealed decreased vacuolar acidity upon longer exposure to haloperidol (Fig. 1B). Quinacrine efficiently labeled the cytoplasm in the presence of haloperidol, suggesting that proton-pumping mechanisms are impaired (Fig. 1B).

### Haloperidol resistance differs between strains and shows transgressive segregation

**Figure 2:**
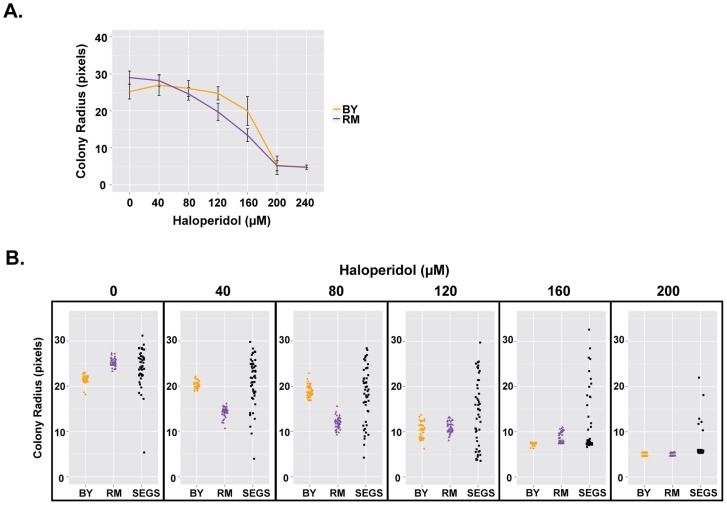
Haloperidol resistance differs between BY and RM, and shows transgressive segregation. (A) Response of BY and RM to haloperidol. Saturated cultures were spotted onto YPD agar plates supplemented with 0–240 µM haloperidol, incubated at 30°C for ∼72 hr. Each strain was replicated 48 times. Mean of colony radius ±1 s.d. are plotted. (B) Haloperidol resistance shows transgressive segregation among progeny from a cross between BY and RM. Cells were grown as in (A), but for 48 hours. 48 replicates of each parent (BY, RM) and 48 random segregants are shown.

We compared growth of BY and RM in the presence of different concentrations of haloperidol (0–240 µM). Although RM has higher baseline growth in the absence of haloperidol, it is more sensitive to haloperidol at concentrations ranging from 40 to 160 µM (Fig. 2A).

We also tested segregants from a cross between BY and RM and found that resistance to haloperidol showed transgressive segregation, with some progeny exhibiting phenotypes more extreme than either parent (Fig. 2B). For instance, haloperidol concentration of 200 µM completely inhibited the growth of both parental strains at 48 hours, but ∼8.5% of segregants were able to grow. A formal statistical test for transgressive segregation [28] showed that it was significant at all measured concentrations of haloperidol between 40 µM and 200 µM (p<0.0001 in all cases; see *Methods* for details).

### Loci underlying haloperidol resistance have dose-dependent effects

**Figure 3:**
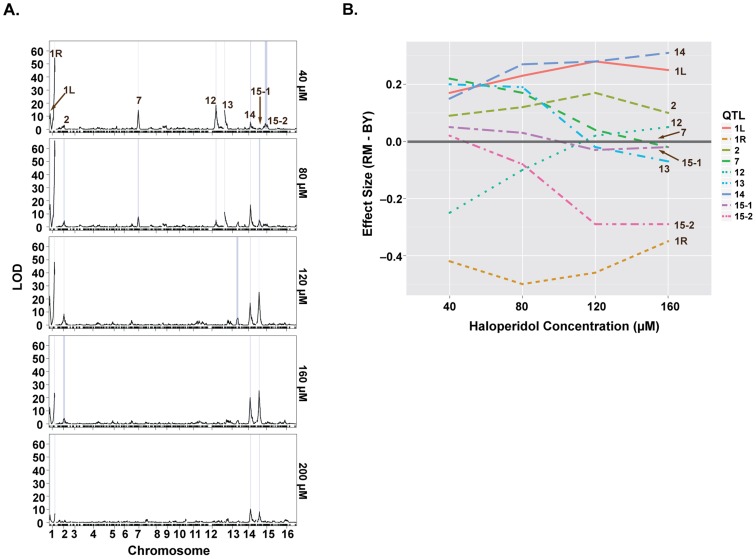
Loci underlying haloperidol resistance have dose-dependent effects. (A) LOD profiles for growth in the presence of increasing doses of haloperidol. Significant loci are shown through their 1.5-LOD drop confidence intervals (blue rectangles). (B) Effect sizes of QTL underlying haloperidol resistance as a function of dose. Effect sizes were calculated via regressing segregant phenotype on genotype (1 versus -1) at a specified locus. Positive values represent better growth in the presence of the BY allele.

We sought to further understand the genetics underlying growth in the presence of haloperidol through QTL mapping. We carried out linkage analysis in a panel of 1008 BY-RM segregants [15] for growth at five different concentrations of haloperidol (40, 80, 120, 160, 200 µM) and identified nine distinct significant QTL (Fig. 3A). At the major locus on the right arm of chromosome I, the allele from RM (the sensitive parent) promoted growth in the presence of haloperidol, consistent with our observation of transgressive segregation (Fig. 2B). RM alleles at loci on chromosomes V, XII and XV also confer greater resistance, while BY alleles confer higher resistance at the remaining five loci (Fig. 3).

Some of the loci were detected only at certain doses of haloperidol, and the effect sizes of most loci were dose-dependent (Fig. 3). For instance, the loci on chromosomes VII, XII, and XIII were only detected at the two lower doses, while the effects of loci on chromosomes XIV and XV primarily manifested at the higher doses (Fig. 3B). Most loci had undetectable or weak effects at 200 µM, because few segregants grew at this dose.

We quantified the amount of variation explained by these nine loci by fitting a linear model with additive QTL. This model explained between 35.9% and 54.7% of the total phenotypic variance at concentrations 40–160 µM (S1 Table). The locus on chromosome I (right arm) alone explained 21.4%, 26.7%, 21.4%, 11.6% of the variance at these four doses, respectively.

### Polymorphisms in the OBP domain of *SWH1* underlie major QTL on chromosome I

The major QTL on the right arm of chromosome I had significant additive effects at all concentrations of haloperidol. The confidence interval of this peak contained the gene *SWH1* (also known as *OSH1*), which encodes a yeast homologue of the mammalian oxysterol-binding protein (OSBP) [29]. OSBPs are a family of proteins with the ability to bind oxysterols [30], [31], which are oxidized derivatives of sterols in the cell.

**Figure 4:**
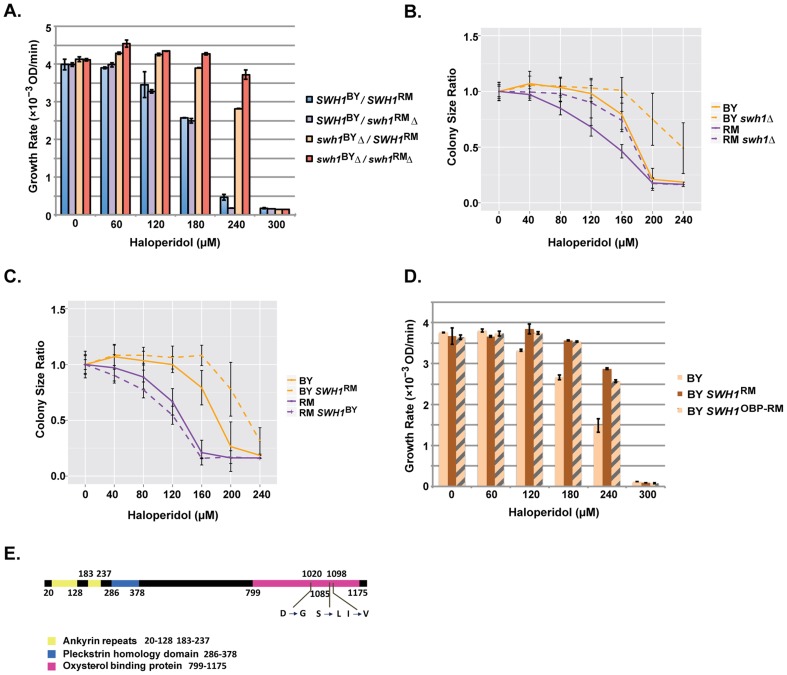
Polymorphisms in the OBP domain of *SWH1* underlie haloperidol resistance. (A) Reciprocal hemizygosity assay as well as double deletion analysis assessing the contribution of *SWH1* and its allelic state in a BY/RM hybrid background. Growth curves were spline fitted to extract the maximum growth rates. Each experiment was performed in triplicate. The mean ±1 s.d. are plotted. (B) Comparison of *swh1Δ* relative to wild type BY and RM. Saturated cultures were spotted onto YPD agar plates supplemented with 0–240 µM haloperidol and plates were incubated at 30°C for ∼72 hr. Shown are colony size ratios obtained by normalizing colony sizes to those on YPD. Mean values ±1 s.d. are plotted. (C) Allele replacements of *SWH1*. Saturated cultures were spotted onto YPD agar plates supplemented with 0–240 µM haloperidol, plated were incubated at 30°C for ∼48 hr. Mean colony size ratio ±1 s.d. are plotted. (D) Replacing the *SWH1* oxysterol binding protein like domain (OBP) in BY with the RM counterpart (OBP-RM) recapitulates the growth rates of replacing the entire *SWH1* gene with the RM allele. (E) Three nonsynonymous SNPs reside in the oxysterol binding protein like domain (OBP) in *SWH1*.

We sequenced the coding region of the BY and RM alleles of *SWH1* and identified 13 synonymous single nucleotide polymorphisms (SNPs), 9 non-synonymous SNPs, and a 6 base pair indel between the two alleles (S2 Table, Fig. 4E). According to Pfam alignments with the amino acid sequence of Osh4 (which recently had its crystal structure solved [32]), Swh1 contains ankyrin repeats, a pleckstrin-homology-protein-like domain (PH), and an oxysterol-binding-protein-like domain (OBP) at the C-terminus [29]. Three of the non-synonymous SNPs between BY and RM are located in the OBP domain, five are in the linker region between the PH domain and OBP domain, and one is in the PH domain (Fig. 4E).

To test whether *SWH1* allelic variation caused differences in growth at different haloperidol concentrations, we conducted a reciprocal hemizygosity analysis [5]. The BY/RM hybrids carrying either only the BY or only the RM allele of *SWH1* grew differently in the presence of haloperidol, demonstrating that *SWH1* contributes to the variable response (Fig. 4A). Specifically, the hybrid carrying only the RM allele of *SWH1* (*swh1^BY^Δ/SWH1* ^RM^) showed a higher growth rate compared to the hybrid carrying only the BY allele (*SWH1^BY^/swh1* ^RM^ *Δ*). Thus, *SWH1* ^RM^ is the resistant allele relative to *SWH1* ^BY^, confirming the QTL results.

We found that deletion of *SWH1* in both BY and RM haploid backgrounds conferred higher growth rates across the response range to haloperidol (Fig. 4B). This illustrates that *SWH1* loss of function leads to greater haloperidol resistance. To gain some insight into the relative function of the BY and RM alleles of *SWH1*, we examined the growth rates of the BY/RM hybrid carrying none, either, or both BY and RM copies of *SWH1* (Fig. 4A). With the BY allele of *SWH1* intact in the hybrid, little difference in growth rate was observed with or without the RM allele, indicating that a single copy of the BY allele of *SWH1* is sufficient for function. Next, comparing the growth rates of the *SWH1* hemizygotes (*swh1^BY^Δ/SWH1* ^RM^ or *SWH1^BY^/swh1* ^RM^ *Δ*) relative to the deletion (*swh1^BY^ Δ*/*swh1^RM^ Δ*), growth of *swh1^BY^Δ/SWH1* ^RM^ was more similar to *swh1^BY^ Δ*/*swh1^RM^ Δ* (Fig. 4A). These results demonstrate that the RM allele of *SWH1* is the less functional of the two. However, the RM allele is not a complete loss-of-function, as deleting the RM allele of *SWH1* still increased haloperidol resistance (Fig. 4B).

Analysis in the BY and RM haploids and their hybrid illustrated that reducing *SWH1* function leads to haloperidol resistance. To explicitly test the effect of *SWH1* polymorphisms on haloperidol resistance, we swapped the *SWH1* coding region in both BY and RM (replacing the coding region with the copy from the other strain). Introducing the functional BY allele into the RM background slightly reduced resistance to haloperidol, whereas having the RM allele of *SWH1* in BY increased resistance. The results from allele replacements in BY and RM haploids demonstrated that *SWH1* affects resistance to haloperidol in both genetic backgrounds (Fig. 4C).

To gain further insight into the mechanism of resistance to haloperidol, we looked more specifically at the polymorphisms between BY and RM in the *SWH1* gene. Among all 22 SNPs residing in the coding region of *SWH1*, three of the non-synonymous SNPs between BY and RM (D1020G, S1085L, and I1098V) are located in the OBP domain (Fig. 4E, S2 Table). We replaced the OBP domain of *SWH1* in BY with the counterpart from RM (hereafter BY *SWH1* ^OBP-RM^) and tested resistance to haloperidol between the replacement strains (Fig. 4D). BY *SWH1* ^OBP-RM^ fully recapitulated the increased resistance to haloperidol achieved by replacing the entire coding region of *SWH1* in BY with the RM allele. According to structure-based alignments of Swh1 with the crystal structure of full length Osh4 in yeast [32], D1020G lies within *β*-sheets (*β*14 - *β*15) that form a hydrophobic tunnel, which can bind one sterol molecule [32]. Therefore, we speculate that D1020G in RM may result in an altered structural form of the binding pocket and reduce Swh1 activity.

### Polymorphisms in *IRA2* and *MKT1* contribute to haloperidol resistance

**Figure 5:**
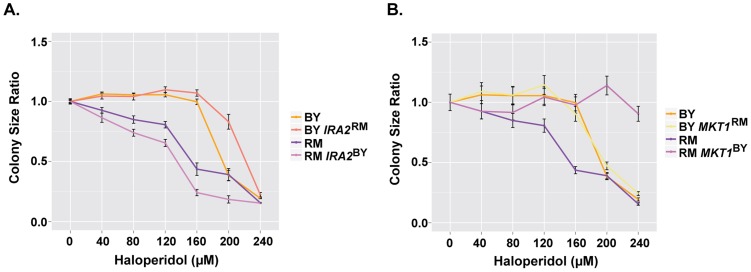
*IRA2* and *MKT1* underlie resistance to haloperidol at high concentrations. (A) Comparison of colony radius ratio between BY and RM with swapped *IRA2* alleles. (B) Comparison of colony radius ratio between BY and RM with swapped *MKT1* alleles. Saturated cultures were spotted onto YPD agar plates supplemented with 0–240 µM haloperidol, plated were incubated at 30°C for ∼48 hr. Mean colony size ratio ±1 s.d. are plotted.

Among the loci identified for haloperidol resistance, those on chromosomes XIV and XV became the major QTL at higher doses of the drug. *IRA2*, a gene previously identified to contain variants underlying differences in gene expression and metabolite levels [33], [34], resides within the locus on chromosome XV. *IRA2* encodes a GTPase activating protein that inhibits RAS, which mediates cellular responses in nutrient limiting conditions via the Ras/PKA pathway [35]–[37]. Analyzing allele replacement strains for *IRA2* [34], we saw that in both parental backgrounds, the allelic state of *IRA2* influenced resistance to haloperidol. The *IRA2* ^RM^ allele in both BY and RM genetic backgrounds was more resistant to the drug (Fig. 5A).

The locus on chromosome XIV is a QTL hotspot identified in many chemical stresses, and as a QTL for growth in rich medium [14], [15], [38]. It contains the gene *MKT1*, which encodes a protein member of a complex involved in HO regulation [39]. A laboratory strain allele of *MKT1* has been shown to influence gene expression [34], DNA replication stress [40] and mitochondrial genome stability [41]. Using BY and RM allele replacement strains from [41], we confirmed *MKT1* as the gene underlying the chromosome XIV locus (Fig. 5B). We further found that *MKT1* only had an effect in the RM background: replacing *MKT1* ^RM^ with *MKT1* ^BY^ led to near complete resistance, while the reciprocal allele swap had little effect on BY (Fig. 5B). The observation that the BY allele of *MKT1* led to greater growth in haloperidol is the opposite of what has been seen for growth in other conditions in previous complex trait studies, which found the *MKT1* ^BY^ allele to be deleterious for growth in the absence of functional mitochondria and in the presence of the drug 4-NQO [9], [41].

### Complex genetic interactions among haloperidol resistance loci

To quantify the amount of phenotypic variance explained by genetic variation, we calculated broad- and narrow-sense heritability [15] at the five concentrations of haloperidol (Table 1). Narrow-sense heritability ranged from 0.56 to 0.71 at doses from 40–160 µM and decreased dramatically at 200 µM. Broad-sense heritability was consistently high (>∼75%) at all doses.

**Table 1:** Broad (H^2^) and narrow (h^2^) sense heritability.

	Haloperidol				
Heritability	40 µM	80 µM	120 µM	160 µM	200 µM
H^2^	0.746±0.039	0.838±0.040	0.897±0.041	0.926±0.042	0.812±0.040
h^2^	0.600±0.084	0.709±0.100	0.696±0.096	0.555±0.086	0.166±0.040
H^2^−h^2^	0.146	0.129	0.201	0.371	0.646
Δvar*	0.110	0.066	0.017	0.078	0.072

* Δvar shows the amount of extra variance explained by incorporating significant QTL pairwise-interactions.

Differences between broad- and narrow-sense heritability suggest that non-additive interactions contribute to phenotypic variance [15]. We tested for statistical interactions between additive QTL detected in at least one haloperidol concentration, and found 9, 10, 3, 5 and 5 significant pair-wise QTL interactions (out of 36 possible locus pairs) at the five doses (Bonferroni-corrected p<0.005, S1 Table, S3 Table). Incorporating the corresponding significant two-way QTL interactions in the QTL model at each dose explained an additional 11.0%, 6.6%, 1.7%, 7.8%, and 7.2% of phenotypic variance at 40 µM, 80 µM, 120 µM, 160 µM, and 200 µM haloperidol, respectively (S3 Table). At 40 µM, the additional variance explained accounted for most of the difference between broad- and narrow-sense heritability (14.6%), and at 80 µM half of this difference (12.9%) was captured (Table 1). However, at higher doses, taking into account two-way interactions explained little of the differences between broad- and narrow-sense heritability (Table 1), suggesting the presence of higher-order interactions, interactions between loci with no detectable main effects, or other non-additive contributions to broad-sense heritability.

We detected significant pair-wise interactions at 160 µM and 200 µM among all pairs of loci on chromosomes I, XIV, and XV that correspond to *SWH1, MKT1*, and *IRA2* (S1 fig., S3 Table). To further explore these interactions, we generated allele replacement strains in both BY and RM carrying all 8 combinations of *SWH1, MKT1*, and *IRA2* alleles (16 total strains), and measured their growth at 200 µM haloperidol. We tested these allelic effects and their interactions using analysis of variance (ANOVA) and found that the pairwise interaction terms were not significant, but all locus pairs had a significant interaction effect with the genetic background (S4 Table). We therefore performed ANOVA in the BY and RM background separately. The pair-wise interactions among *SWH1*, *MKT1*, and *IRA2* were all significant in the RM background, but none were significant in the BY background (Table 2, S4, S5, and S6 Table).

**Table 2:** Effects of *SWH1, MKT1, IRA2* genes and their interactions on growth in haloperidol (RM background, 200 µM).

Coefficients	Estimate	Std. Error	t value	Pr(>|t|)
(Intercept)	0.40242	0.02972	13.539	<2e-16
*MKT1*(BY)	−0.12212	0.04203	−2.905	0.00396
*IRA2*(RM)	−0.32085	0.04203	−7.633	3.62e-13
*SWH1*(RM)	−0.27910	0.04203	−6.640	1.63e-10
*MKT1*(BY): *IRA2*(RM)	0.24819	0.05944	4.175	3.98e-05
*MKT1*(BY): *SWH1*(RM)	0.77430	0.05944	13.026	<2e-16
*IRA2*(RM): *SWH1*(RM)	0.26932	0.05944	4.531	8.72e-06
*MKT1*(BY): *IRA2*(RM): *SWH1*(RM)	0.07578	0.08407	0.901	0.36812

**Figure 6:**
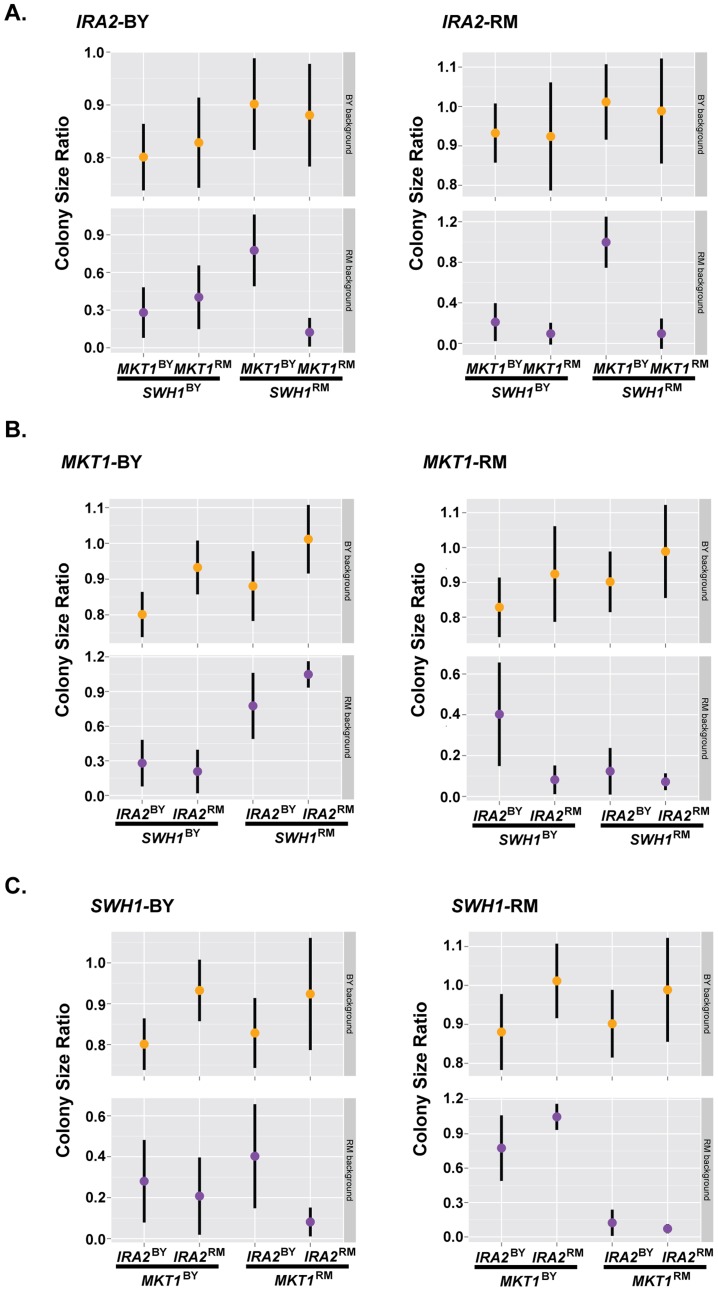
Complex genetic interactions underlie resistance to haloperidol. Growth of strains carrying all possible permutations of BY and RM alleles of the genes *MKT1, IRA2*, and *SWH1* in the BY and RM backgrounds grouped (A) by their *IRA2* genotypes, (B) by their *MKT1* genotypes, and (C) by their *SWH1* genotypes. 36 replicates of each strain were spotted onto agar supplemented with 200 µM haloperidol. Dots show the mean, vertical bars show 1 standard deviation.

These results suggest that complex interactions among the three tested alleles (*SWH1, MKT1, IRA2*) and the genetic background determine resistance to haloperidol. In the RM background, the allelic state of *SWH1* influenced the effect of *MKT1*. Introduction of *MKT1* ^BY^ dramatically increased growth, but only in the presence of *SWH1* ^RM^ (Fig. 6A). We also found interactions between the alleles of *SWH1* and *IRA2* in the RM background (Fig. 6B). *IRA2* ^RM^ increased growth in RM when it carried *SWH1* ^RM^ and *MKT1* ^BY^ (Fig. 6B, left panel), but reduced growth when it carried *SWH1* ^BY^ and *MKT1* ^RM^ (Fig. 6, right panel). Further, the allelic state of *SWH1* influenced the direction of effect for *IRA2. IRA2* ^BY^ promoted growth in the presence of the BY allele of *SWH1* (Fig. 6C, left panel), but reduced growth in the presence of the RM allele of *SWH1* (Fig. 6C, right panel).

Growth of both BY and RM in haloperidol was completely rescued by the genotype combination *MKT1* ^BY^, *IRA2* ^RM^ and *SWH1* ^RM^ (Fig. 6). However, *MKT1* ^RM^ only caused sensitivity to haloperidol in RM (Fig. 6B, right panel), even in the presence of *SWH1* ^RM^ and *IRA2* ^RM^. We therefore conclude that *MKT1* ^RM^, in combination with other unidentified factors in the RM background contribute to the sensitivity of RM to haloperidol.

## Discussion

The genetic architectures of chemical resistance in yeast range from relatively simple (involving a single locus) to highly complex (>20 loci) [9], [13]–[15]. These studies typically tested only one dose per compound. Here, we explored the full dose response range of the small molecule drug haloperidol to dissect the genetic architecture of dose-response variation in *S. cerevisiae*. We have shown that loci underlying haloperidol resistance have dose-dependent effects. We identified QTL that showed effects only at low doses of haloperidol, and loci that showed significant effects primarily at higher concentrations of the drug. Our study demonstrates QTL-dosage interaction within the response range of a single drug, and provides new insight into the complex genetic basis of drug resistance in yeast.

We identified *SWH1* (*OSH1*) to be the causal gene underlying the largest effect locus in response to haloperidol. Swh1 is a protein similar to the mammalian oxysterol-binding protein and targets to both the Golgi and the nucleus-vacuole junction in yeast [42]. Swh1 that associates with the nucleus-vacuole junction has been shown to act as a substrate for a degradation process, named the piecemeal microautophagy of the nucleus (PMN) [43]. Our observation that variants within the OBP domain of Swh1 contribute to resistance to haloperidol suggests that cellular transport, perhaps of sterol-related molecules, is affected in the presence of haloperidol. Cationic amphiphilic drugs have been linked to phospholipidosis and cellular membrane damage [19], and our identification of Swh1 suggests a potential role for oxysterol binding proteins in these defects. We found that after 6 hours of exposure to haloperidol, yeast vacuoles were enlarged, with the cytoplasm more acidic than the vacuoles, suggesting that haloperidol leads to vacuole dysfunction and further linking Swh1, vacuole functions, and haloperidol resistance. The same locus was previously linked to growth in E6 berbamine, cobalt chloride, copper sulphate, and neomycin [13], [15]; it also overlaps with a QTL hotspot in response to a panel of small-molecule therapeutic drugs [13], suggesting that this locus has pleiotropic effects.

In *S. cerevisiae*, there are seven OSBP homologues (*OSH1-7*) [44]. Previous studies of the yeast *OSH* genes suggested that the seven oxysterol-binding proteins shared at least one essential role in the cell (only deletion of all seven genes is lethal), and their functions have significant overlap [44]. We have here provided genetic evidence that Swh1 functions are related to resistance to haloperidol. BY and RM display variation in both the coding and non-coding regions of the remaining six *OSH* genes. These six *OSH* genes do not lie in the detected QTL intervals, suggesting that the variants within these genes may lack effects on growth in the presence of haloperidol, either because they do not alter gene function or because only *SWH1* has an effect on growth in the presence of haloperidol. Further studies are required to tease apart the specific functions of the individual yeast *OSH* genes.

We showed that polymorphisms in *MKT1* contribute to yeast growth in the presence of high concentrations of haloperidol. *MKT1* is also a hotspot identified in eQTL [34], protein QTL [45]–[47], and drug resistance studies [13] in yeast. The BY (isogenic derivative of S288c) allele of *MKT1*, which is not present in other strain backgrounds, was previously shown to reduce formation of *petite* colonies and compromise growth of *petite* cells [41]. Lipophilic cations can pass through phospholipid membranes, especially those with a large transmembrane potential, such as the mitochondrial inner membrane. This leads to the accumulation of these drugs in the mitochondrial matrix, inducing mitochondrial respiration inhibition [48]. The observation that the BY allele of *MKT1* confers resistance to haloperidol suggests that haloperidol may compromise mitochondrial integrity. Variants in *IRA2* also contribute to haloperidol resistance. The RM allele of *IRA2* inhibits the Ras/PKA pathway more strongly than the BY allele [34]. Since PKA inhibits Msn2/Msn4, the major transcription factors in stress response [49], [50], the RM allele of *IRA2* is predicted to lead to stronger stress response, suggesting that stronger stress response may be advantageous at high haloperidol concentrations.

In this study, we demonstrated complex interactions among the alleles of *SWH1*, *MKT1*, and *IRA2* in the RM background at 200 µM haloperidol. Pair-wise interactions between identified loci explained the majority of the difference between broad- and narrow-sense heritability at 40 µM haloperidol, but not at higher doses, suggesting higher order interactions or other non-additive contributions. Previous studies in yeast using sporulation efficiency as a model for complex traits [10], [11] revealed linkage between small- and large-effect QTL, as well as interactions among these QTL. Small-effect QTL were found to depend on the allelic status of the large-effect QTL [11], which is similar to our observation that the effects of *IRA2* and *MKT1* were dependent on the allele of *SWH1* – the gene underlying the large effect QTL. Through allele replacement analyses, we found that the interactions between *SWH1*, *MKT1*, and *IRA2* were present in the RM background but absent in the BY background, illustrating the value of studying genetically diverse strains.

Haloperidol and many antidepressants are cationic amphiphilic drugs that accumulate in membranes in the absence of their specific targets [51]. *SWH1* is functionally related to sterol trafficking and the membrane system, and underlies the QTL detected throughout the entire dose response in haloperidol. The identification of *MKT1* and *IRA2* at higher concentrations of haloperidol suggests the effects of other cellular processes and stress responses. Given the current knowledge on the functions of these identified genes, the interactions between *SWH1, MKT1*, and *IRA2* could reflect underlying mechanisms that connect the membrane system, sterol metabolism, and stress response. The as yet unidentified genes underlying the remaining QTL may provide further insight into the mechanisms of action of haloperidol.

## Materials and Methods

### Yeast strains, media, and chemicals

*S. cerevisiae* strains BY4724 and RM11-1a derived strains were used in this study. The panel of 1008 prototrophic segregants derived from BY (MATa) and RM (MAT**α** *hoΔ*::HphMX4 *flo8Δ*::NatMX4 *AMN1* ^BY^) was previously generated [15]. Allele replacement strains were constructed via the *Delitto Perfetto* approach using the CORE cassette [52]. This two-step process was performed by first inserting a *URA3-*KanMX4 cassette from pCORE to generate *yfgΔ*::*URA3-*KanMX4; then the region of interest was amplified through high-fidelity PCR from the donor strain, and inserted to replace the *URA3-*KanMX4 cassette. The loss of the *URA3-*KanMX4 cassette was selected via 5-Fluoroorotic Acid (5-FOA) counter selection of *URA3* and further selected via loss of G418 resistance. Single colonies were isolated at each step, cassette insertions were confirmed via PCR, and allele replacements were sequenced to verify the presence of the correct allele. Transformations were performed by the standard lithium acetate method. All gene sequences were obtained from the *Saccharomyces* Genome Database (http://www.yeastgenome.org/). All DNA sequencing related to strain construction and confirmation was performed through standard dideoxy methods.

Cultures were grown in rich medium (YPD, 1% yeast extract, 2% peptone and 2% glucose). YPD liquid media and agar plates were made as described [53]. SPO++ was used for sporulation (http://www.genomics.princeton.edu/dunham/sporulationdissection.htm). Selection plates for strain construction were made with YPD containing the respective drugs at standard doses. Haloperidol was purchased from Sigma (Sigma H1512). All drugs in this study are dissolved in DMSO. Because BY and RM exhibit growth defects only at DMSO concentrations >3% (v/v%), DMSO concentrations in all experiments were kept at <1%.

### Selection agar plate construction

Drug selection agar plates were made with Nunc OmniTray (Thermo Scientific 264728). 50 mL of YPD with drug concentrations specified were poured into each tray, the trays were placed on a flat surface to solidify in order to obtain best pinning results. All trays contained the same final DMSO concentration. Each experiment was performed with the same batch of YPD. Each plate was made 6 times to allow testing 2 full replicates of the entire segregant panel in 2 different layout configurations. Segregants were pinned on to each agar tray in 384 well format. Haloperidol concentrations for agar plates were selected to be 40, 80, 120, 160, and 200 µM. These concentrations capture the growth differences between BY and RM, yet maintain enough colony growth to allow QTL mapping.

### Yeast colony growth measurement

The 1008 segregant panel are stored at −80°C in 96 well format. They were inoculated in YPD and cultured in 384-well plates for ∼48 hours or until saturation. Two configurations were used when converting from 96 to 384 well format, to control for position effect and growth differences due to neighboring cells (including blank controls). Culture plates were then fully resuspended and pinned onto corresponding agar plates with 384 long pins using Singer RoToR. The pinned agar plates were incubated at 30°C for 48–72 hr (as specified) and scanned with an Epson 700 transparency scanner. TIFF images (400 dpi) were processed for end-point colony size and effective colony radius was used as proxy for growth [15].

### QTL mapping

In order to control for both intrinsic growth rate differences and plate position effects, end point effective colony radiuses (as described in “Yeast colony growth measurement”) were normalized for growth on control media (YPD supplemented with same amounts of DMSO as solvent control) through fitting a regression for effect of growth that were in the same layout configuration on YPD. Residuals were used for QTL mapping. Linkage was determined by calculating LOD scores for each genotype marker using both Haley-Knott regression and non-parametric linkage mapping with the R/qtl package. QTL were called at a LOD cutoff of 3. Significance was further determined by 1000 permutations of phenotypic values, and re-calculation of LOD scores.

### Growth rate quantification

Yeast cells were inoculated in rich medium in 96-well plates (Costar 3370) and incubated at 30°C until saturation. 1% (v/v%) of saturated culture was used in fresh medium (with or without drug) for growth rate measurement (starting optical density OD <0.05). Growth curves were recorded using Synergy 2 Multi-Mode Microplate Reader (BioTek Instruments) at 30°C with continuous fast linear shaking (100 µL/well). OD_600_ were collected at 15-minute intervals for up to 24 hours. Growth curves were spline fitted, and the maximum fitted slope during logarithmic phase was used as maximum growth rate. Each strain/condition was performed in triplicate. Growth rates are shown as the mean ± sstandard deviation. A t-test was performed between samples in comparison to obtain p-values. All data fitting and comparison were performed in R (http://www.r-project.org/).

### Replacement analysis

Thirty-six replicates each of sixteen replacement strains (BY background: *SWH1* ^RM^, *MKT1* ^RM^, *IRA2* ^RM^, *IRA2* ^RM^ *SWH1* ^RM^, *IRA2* ^RM^ *MKT1* ^RM^, *SWH1* ^RM^ *MKT1* ^RM^, *SWH1* ^RM^ *IRA2* ^RM^ *MKT1* ^RM^; RM background: *SWH1* ^BY^, *MKT1* ^BY^, *IRA2* ^BY^, *MKT1* ^BY^ *SWH1* ^BY^, *MKT1* ^BY^ *IRA2* ^BY^, *SWH1* ^BY^ *IRA2* ^BY^, *SWH1* ^BY^ *MKT1* ^BY^ *IRA2* ^BY^) and wild type progenitor BY and RM strains were spotted onto YPD agar supplemented with 0, 40, 80, 120, 160, 200 µM haloperidol. Plates were incubated at 30°C for ∼72 hr, then scanned as described above in “Yeast colony growth measurement”. Colony radiuses were extracted after image processing. The effect of replacements or replacement combinations was compared to their otherwise isogenic progenitor through analysis of variance (ANOVA). The analysis was conducted in R.

### Transgressive test

The test for transgression was adapted from [28]. Briefly, segregants and parents were tabulated, and the pooled variance was calculated. Segregants that were 2 standard deviations above the mean of the high parent or below the mean of the low parent were counted. The null model was constructed by pooling the segregants and parents, and the null parents and null segregants were randomly sampled from this pool. Significance was determined based on resampling 10,000 times the pooled null model.

### Vacuole fluorescent staining and microscopy

Yeast cells in early to mid log-phase were divided into two cultures of equal volume. Haloperidol (final concentration 150 µM) was added to one half, while an equal volume of DMSO as solvent control was added to the other half. The cultures were incubated at 30°C on a shaker for ∼2 hr. Then, 2×10^6^ cells were harvested from each culture for subsequent staining.

Quinacrine (Sigma Q3251) staining of vacuoles was performed as described in [54]. Harvested cells were washed once in buffered YPD (supplemented with 100 mM HEPES, pH 7.6), resuspended in 100 µL of the same buffered medium and quinacrine at a final concentration of 200 µM. Cell suspensions were incubated at 30°C for 10min and placed on ice for 5min. Cells were pelleted, washed twice, resuspended with ice-old 100 mM HEPES, pH 7.6 buffer containing 2% glucose and kept on ice until imaging.

Carboxy-DCFDA (Yeast Vacuole Marker Sampler Kit, Molecular Probes Y-7531) staining was performed according to kit instructions. Briefly, harvested cells were washed and resuspended in 50 mM sodium citrate buffer, pH 5.0, containing 2% glucose. Carboxy-DCFDA at a final concentration of 10 µM was added to the cell suspension followed by incubation at room temperature for 15–30min.

For FM4-64 (Molecular Probes, T-3166) staining [55], harvested cells were resuspended in 50 µL YPD with 1 µL FM4-64 stock solution (1.6 µM in DMSO) and incubated at 30°C for 20min. Cells were washed subsequently with 1 mL YPD at room temperature and resuspended in 5 mL YPD to recover at 30°C on a shaker for 90–120min. Recovered cells were washed once in 1 mL sterile ddH_2_O and resuspended in 200–500 µL YNB for imaging.

All imaging was performed within 30min of staining on an Olympus IX81 inverted fluorescence microscope [56] using a 100× oil objective. Quinacrine and carboxy-DCFDA staining were visualized with Chroma SP101v2 (FITC), and FM4-64 with Chroma 49008 (mCherry TexasRed) filter sets. Images were acquired using Slidebook 5.0 digital image acquisition software (Intelligent Imaging Innovations) and processed using ImageJ version 1.46r.

## Supporting Information

S1 Figure Interactions between loci on chromosomes I, XIV, and XV at 160 µM haloperidol. 1008 segregants were grouped based on their genotypes at the above loci, and phenotype means for each group ±1 s.e. are plotted. (A) Interaction between QTL on chromosomes I and XIV; (B) Interaction between QTL on chromosomes I and XV; (C) Interaction between QTL on chromosomes XIV and XIV.
(TIF)

S1 Table QTL model with additive loci and variance estimates (Drop one QTL at a time ANOVA table).
(DOCX)

S2 Table Polymorphisms in the coding region of gene SWH1 (YAR042W) between BY and RM.
(DOCX)

S3 Table QTL models incorporating significant two-way QTL interactions (drop one QTL at a time ANOVA tables).
(DOCX)

S4 Table Effects of SWH1, MKT1, IRA2 genes, the genetic background (BG) and their interactions on growth in haloperidol (200 µM).
(DOCX)

S5 Table Effects of SWH1, MKT1, IRA2 genes and their interactions on growth in haloperidol (BY background, 200 µM).
(DOCX)

S6 Table Tukey-HSD test of SWH1, MKT1, and IRA2 allelic effects, and their two-way interactions (200 µM haloperidol).
(DOCX)

## References

[1] Liti G, Louis EJ (2012) Advances in Quantitative Trait Analysis in Yeast. Plos Genetics 8: e1002912. 22916041 10.1371/journal.pgen.1002912 PMC3420948

[2] Brem RB, Yvert G, Clinton R, Kruglyak L (2002) Genetic dissection of transcriptional regulation in budding yeast. Science 296: 752–755. 11923494 10.1126/science.1069516

[3] Fay JC, McCullough HL, Sniegowski PD, Eisen MB (2004) Population genetic variation in gene expression is associated with phenotypic variation in Saccharomyces cerevisiae. Genome Biology 5: R26. 15059259 10.1186/gb-2004-5-4-r26 PMC395785

[4] Picotti P, Clement-Ziza M, Lam H, Campbell DS, Schmidt A, et al (2013) A complete mass-spectrometric map of the yeast proteome applied to quantitative trait analysis. Nature 494: 266–270. 23334424 10.1038/nature11835 PMC3951219

[5] Steinmetz LM, Sinha H, Richards DR, Spiegelman JI, Oefner PJ, et al (2002) Dissecting the architecture of a quantitative trait locus in yeast. Nature 416: 326–330. 11907579 10.1038/416326a

[6] Sinha H, David L, Pascon RC, Clauder-Munster S, Krishnakumar S, et al (2008) Sequential elimination of major-effect contributors identifies additional quantitative trait loci conditioning high-temperature growth in yeast. Genetics 180: 1661–1670. 18780730 10.1534/genetics.108.092932 PMC2581965

[7] Cubillos FA, Parts L, Salinas F, Bergstrom A, Scovacricchi E, et al.. (2013) High-Resolution Mapping of Complex Traits with a Four-Parent Advanced Intercross Yeast Population. Genetics 195: 1141-+. 10.1534/genetics.113.155515 PMC3813843 24037264

[8] Yang YD, Foulquie-Moreno MR, Clement L, Erdei E, An TH, et al (2013) QTL Analysis of High Thermotolerance with Superior and Downgraded Parental Yeast Strains Reveals New Minor QTLs and Converges on Novel Causative Alleles Involved in RNA Processing. Plos Genetics 9: e1003693. 23966873 10.1371/journal.pgen.1003693 PMC3744412

[9] Demogines A, Smith E, Kruglyak L, Alani E (2008) Identification and dissection of a complex DNA repair sensitivity phenotype in Baker's yeast. PLoS Genet 4: e1000123. 18617998 10.1371/journal.pgen.1000123 PMC2440805

[10] Gerke J, Lorenz K, Cohen B (2009) Genetic interactions between transcription factors cause natural variation in yeast. Science 323: 498–501. 19164747 10.1126/science.1166426 PMC4984536

[11] Lorenz K, Cohen BA (2012) Small- and large-effect quantitative trait locus interactions underlie variation in yeast sporulation efficiency. Genetics 192: 1123–1132. 22942125 10.1534/genetics.112.143107 PMC3522155

[12] Deutschbauer AM, Davis RW (2005) Quantitative trait loci mapped to single-nucleotide resolution in yeast. Nature Genetics 37: 1333–1340. 16273108 10.1038/ng1674

[13] Perlstein EO, Ruderfer DM, Roberts DC, Schreiber SL, Kruglyak L (2007) Genetic basis of individual differences in the response to small-molecule drugs in yeast. Nat Genet 39: 496–502. 17334364 10.1038/ng1991

[14] Ehrenreich IM, Torabi N, Jia Y, Kent J, Martis S, et al (2010) Dissection of genetically complex traits with extremely large pools of yeast segregants. Nature 464: 1039–1042. 20393561 10.1038/nature08923 PMC2862354

[15] Bloom JS, Ehrenreich IM, Loo WT, Lite TL, Kruglyak L (2013) Finding the sources of missing heritability in a yeast cross. Nature 494: 234–237. 23376951 10.1038/nature11867 PMC4001867

[16] Sumiyoshi T, Kido H, Sakamoto H, Urasaki K, Suzuki K, et al (1994) In vivo dopamine-D2 and serotonin-5-HT2 receptor binding study of risperidone and haloperidol. Pharmacol Biochem Behav 47: 553–557. 7516078 10.1016/0091-3057(94)90158-9

[17] Ericson E, Gebbia M, Heisler LE, Wildenhain J, Tyers M, et al (2008) Off-target effects of psychoactive drugs revealed by genome-wide assays in yeast. PLoS Genet 4: e1000151. 18688276 10.1371/journal.pgen.1000151 PMC2483942

[18] Rainey MM, Korostyshevsky D, Lee S, Perlstein EO (2010) The antidepressant sertraline targets intracellular vesiculogenic membranes in yeast. Genetics 185: 1221–1233. 20457874 10.1534/genetics.110.117846 PMC2927751

[19] Halliwell WH (1997) Cationic amphiphilic drug-induced phospholipidosis. Toxicol Pathol 25: 53–60. 9061852 10.1177/019262339702500111

[20] Chen J, Korostyshevsky D, Lee S, Perlstein EO (2012) Accumulation of an antidepressant in vesiculogenic membranes of yeast cells triggers autophagy. PLoS One 7: e34024. 22529904 10.1371/journal.pone.0034024 PMC3329523

[21] Casey DR, Sebai SC, Shearman GC, Ces O, Law RV, et al (2008) Formulation affects the rate of membrane degradation catalyzed by cationic amphiphilic drugs. Industrial & Engineering Chemistry Research 47: 650–655.

[22] Lum PY, Armour CD, Stepaniants SB, Cavet G, Wolf MK, et al (2004) Discovering modes of action for therapeutic compounds using a genome-wide screen of yeast heterozygotes. Cell 116: 121–137. 14718172 10.1016/s0092-8674(03)01035-3

[23] Moebius FF, Bermoser K, Reiter RJ, Hanner M, Glossmann H (1996) Yeast sterol C8-C7 isomerase: identification and characterization of a high-affinity binding site for enzyme inhibitors. Biochemistry 35: 16871–16878. 8988026 10.1021/bi961996m

[24] Jones EW, Pringle JR, Broach JR (1992) The Molecular and Cellular Biology of the Yeast Saccharomyces: Cold Spring Harbor Laboratory Press.

[25] Zweytick D, Hrastnik C, Kohlwein SD, Daum G (2000) Biochemical characterization and subcellular localization of the sterol C-24(28) reductase, erg4p, from the yeast saccharomyces cerevisiae. FEBS Lett 470: 83–87. 10722850 10.1016/s0014-5793(00)01290-4

[26] Olcer M, Hakyemez G (1988) Investigations of Some Physicochemical Properties of Haloperidol Which May Affect Its Activity. Journal of Clinical Pharmacy and Therapeutics 13: 341–349. 3230099 10.1111/j.1365-2710.1988.tb00203.x

[27] Banta LM, Robinson JS, Klionsky DJ, Emr SD (1988) Organelle assembly in yeast: characterization of yeast mutants defective in vacuolar biogenesis and protein sorting. J Cell Biol 107: 1369–1383. 3049619 10.1083/jcb.107.4.1369 PMC2115260

[28] Brem RB, Kruglyak L (2005) The landscape of genetic complexity across 5,700 gene expression traits in yeast. Proceedings of the National Academy of Sciences of the United States of America 102: 1572–1577. 15659551 10.1073/pnas.0408709102 PMC547855

[29] Schmalix WA, Bandlow W (1994) SWH1 from yeast encodes a candidate nuclear factor containing ankyrin repeats and showing homology to mammalian oxysterol-binding protein. Biochim Biophys Acta 1219: 205–210. 8086466 10.1016/0167-4781(94)90273-9

[30] Taylor FR, Saucier SE, Shown EP, Parish EJ, Kandutsch AA (1984) Correlation between Oxysterol Binding to a Cytosolic Binding-Protein and Potency in the Repression of Hydroxymethylglutaryl Coenzyme-a Reductase. Journal of Biological Chemistry 259: 2382–2387. 6490619

[31] Dawson PA, Vanderwesthuyzen DR, Goldstein JL, Brown MS (1989) Purification of Oxysterol Binding-Protein from Hamster Liver Cytosol. Journal of Biological Chemistry 264: 9046–9052. 2722817

[32] Im YJ, Raychaudhuri S, Prinz WA, Hurley JH (2005) Structural mechanism for sterol sensing and transport by OSBP-related proteins. Nature 437: 154–158. 16136145 10.1038/nature03923 PMC1431608

[33] Breunig JS, Hackett SR, Rabinowitz JD, Kruglyak L (2014) Genetic basis of metabolome variation in yeast. PLoS Genet 10: e1004142. 24603560 10.1371/journal.pgen.1004142 PMC3945093

[34] Smith EN, Kruglyak L (2008) Gene-environment interaction in yeast gene expression. PLoS Biol 6: e83. 18416601 10.1371/journal.pbio.0060083 PMC2292755

[35] Tanaka K, Lin BK, Wood DR, Tamanoi F (1991) Ira2, an Upstream Negative Regulator of Ras in Yeast, Is a Ras Gtpase-Activating Protein. Proceedings of the National Academy of Sciences of the United States of America 88: 468–472. 1988946 10.1073/pnas.88.2.468 PMC50832

[36] Tanaka K, Nakafuku M, Tamanoi F, Kaziro Y, Matsumoto K, et al (1990) Ira2, a 2nd Gene of Saccharomyces-Cerevisiae That Encodes a Protein with a Domain Homologous to Mammalian Ras Gtpase-Activating Protein. Molecular and Cellular Biology 10: 4303–4313. 2164637 10.1128/mcb.10.8.4303-4313.1990 PMC360976

[37] Broach JR (1991) Ras Genes in Saccharomyces-Cerevisiae - Signal Transduction in Search of a Pathway. Trends in Genetics 7: 28–33. 1848378 10.1016/0168-9525(91)90018-l

[38] Ehrenreich IM, Bloom J, Torabi N, Wang X, Jia Y, et al (2012) Genetic architecture of highly complex chemical resistance traits across four yeast strains. PLoS Genet 8: e1002570. 22438822 10.1371/journal.pgen.1002570 PMC3305394

[39] Tadauchi T, Inada T, Matsumoto K, Irie K (2004) Posttranscriptional regulation of HO expression by the Mkt1-Pbp1 complex. Mol Cell Biol 24: 3670–3681. 15082763 10.1128/MCB.24.9.3670-3681.2004 PMC387745

[40] Tkach JM, Yimit A, Lee AY, Riffle M, Costanzo M, et al (2012) Dissecting DNA damage response pathways by analysing protein localization and abundance changes during DNA replication stress. Nature Cell Biology 14: 966–976. 22842922 10.1038/ncb2549 PMC3434236

[41] Dimitrov LN, Brem RB, Kruglyak L, Gottschling DE (2009) Polymorphisms in multiple genes contribute to the spontaneous mitochondrial genome instability of Saccharomyces cerevisiae S288C strains. Genetics 183: 365–383. 19581448 10.1534/genetics.109.104497 PMC2746160

[42] Levine TP, Munro S (2001) Dual targeting of Osh1p, a yeast homologue of oxysterol-binding protein, to both the Golgi and the nucleus-vacuole junction. Molecular Biology of the Cell 12: 1633–1644. 11408574 10.1091/mbc.12.6.1633 PMC37330

[43] Kvam E, Goldfarb DS (2004) Nvj1p is the outer-nuclear-membrane receptor for oxysterol-binding protein homolog Osh1p in Saccharomyces cerevisiae. Journal of Cell Science 117: 4959–4968. 15367582 10.1242/jcs.01372

[44] Beh CT, Cool L, Phillips J, Rine J (2001) Overlapping functions of the yeast oxysterol-binding protein homologues. Genetics 157: 1117–1140. 11238399 10.1093/genetics/157.3.1117 PMC1461579

[45] Foss EJ, Radulovic D, Shaffer SA, Goodlett DR, Kruglyak L, et al (2011) Genetic Variation Shapes Protein Networks Mainly through Non-transcriptional Mechanisms. Plos Biology 9: e1001144. 21909241 10.1371/journal.pbio.1001144 PMC3167781

[46] Khan Z, Bloom JS, Garcia BA, Singh M, Kruglyak L (2009) Protein quantification across hundreds of experimental conditions. Proceedings of the National Academy of Sciences of the United States of America 106: 15544–15548. 19717460 10.1073/pnas.0904100106 PMC2732709

[47] Albert FW, Treusch S, Shockley AH, Bloom JS, Kruglyak L (2014) Genetics of single-cell protein abundance variation in large yeast populations. Nature 506: 494–497. 24402228 10.1038/nature12904 PMC4285441

[48] Dykens JA, Will Y (2008) Drug-induced mitochondrial dysfunction. Hoboken, NJ: John Wiley & Sons xvii, 616 p., 612 p., of plates p

[49] Boy-Marcotte E, Perrot M, Bussereau F, Boucherie H, Jacquet M (1998) Msn2p and Msn4p control a large number of genes induced at the diauxic transition which are repressed by cyclic AMP in Saccharomyces cerevisiae. Journal of Bacteriology 180: 1044–1052. 9495741 10.1128/jb.180.5.1044-1052.1998 PMC106990

[50] Schmitt AP, McEntee K (1996) Msn2p, a zinc finger DNA-binding protein, is the transcriptional activator of the multistress response in Saccharomyces cerevisiae. Proceedings of the National Academy of Sciences of the United States of America 93: 5777–5782. 8650168 10.1073/pnas.93.12.5777 PMC39137

[51] Goldstein DB (1984) The effects of drugs on membrane fluidity. Annu Rev Pharmacol Toxicol 24: 43–64. 6329077 10.1146/annurev.pa.24.040184.000355

[52] Storici F, Lewis LK, Resnick MA (2001) In vivo site-directed mutagenesis using oligonucleotides. Nat Biotechnol 19: 773–776. 11479573 10.1038/90837

[53] Burke D, Dawson D, Stearns T (2000) Methods in Yeast Genetics: A Cold Spring Harbor Laboratory Course Manual: CSHL Press.

[54] Morano KA, Klionsky DJ (1994) Differential effects of compartment deacidification on the targeting of membrane and soluble proteins to the vacuole in yeast. J Cell Sci 107 (Pt 10): 2813–2824. 10.1242/jcs.107.10.2813 7876349

[55] Vida TA, Emr SD (1995) A new vital stain for visualizing vacuolar membrane dynamics and endocytosis in yeast. J Cell Biol 128: 779–792. 7533169 10.1083/jcb.128.5.779 PMC2120394

[56] Silverman SJ, Petti AA, Slavov N, Parsons L, Briehof R, et al (2010) Metabolic cycling in single yeast cells from unsynchronized steady-state populations limited on glucose or phosphate. Proc Natl Acad Sci U S A 107: 6946–6951. 20335538 10.1073/pnas.1002422107 PMC2872461

