# Supplementary figures and images for: Genetic Basis of Haloperidol Resistance in Saccharomyces cerevisiae Is Complex and Dose Dependent
Source: PLoS Genet. 2014 Dec 18;10(12):e1004894. doi: 10.1371/journal.pgen.1004894 (PMC4270474; doi:10.1371/journal.pgen.1004894)

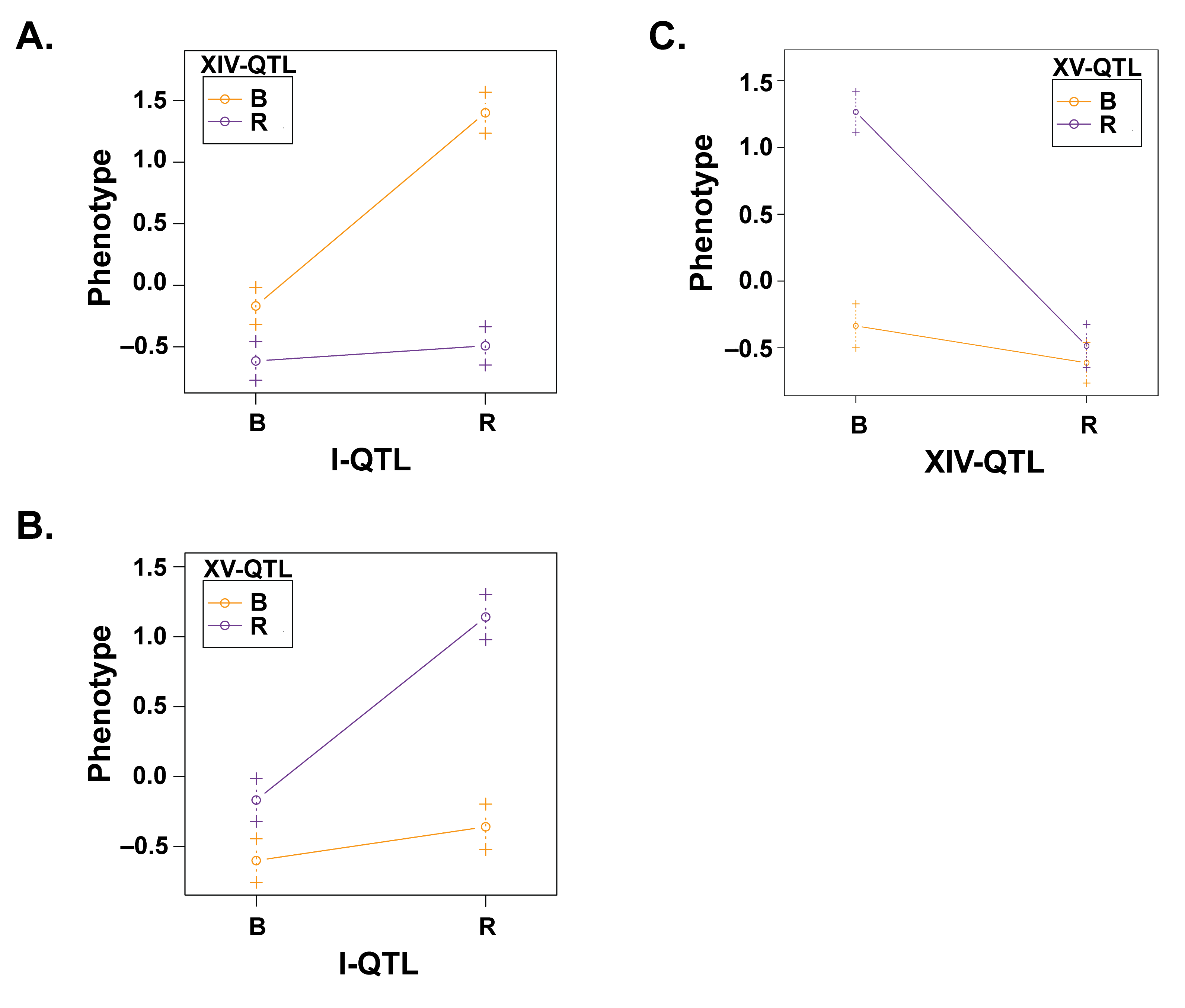

Supplement: S1 Figure — Interactions between loci on chromosomes I, XIV, and XV at 160 µM haloperidol. 1008 segregants were grouped based on their genotypes at the above loci, and phenotype means for each group ±1 s.e. are plotted. (A) Interaction between QTL on chromosomes I and XIV; (B) Interaction between QTL on chromosomes I and XV; (C) Interaction between QTL on chromosomes XIV and XIV. (TIF) [file pgen.1004894.s001.tif]
