# Supplementary material for: Genetic Basis of Haloperidol Resistance in Saccharomyces cerevisiae Is Complex and Dose Dependent
Source: PLoS Genet. 2014 Dec 18;10(12):e1004894. doi: 10.1371/journal.pgen.1004894 (PMC4270474; doi:10.1371/journal.pgen.1004894)
Supplement: S1 Table — QTL model with additive loci and variance estimates (Drop one QTL at a time ANOVA table). (DOCX) [file pgen.1004894.s002.docx]

**Table S1. QTL model with additive loci and variance estimates (Drop one QTL at a time ANOVA table).**

40 μM haloperidol:

|  | df | Type III SS | LOD | %var | F value | Pvalue(Chi2) | Pvalue(F) |
| --- | --- | --- | --- | --- | --- | --- | --- |
| 1L | 1 | 1047.8 | 17.654 | 4.2752 | 83.95 | 0.000 | < 2e-16 |
| 1R | 1 | 5231.9 | 76.554 | 21.3477 | 419.18 | 0.000 | < 2e-16 |
| 2 | 1 | 174.5 | 3.043 | 0.7121 | 13.98 | 0.000 | 0.000195 |
| 7 | 1 | 1199.2 | 20.092 | 4.8933 | 96.08 | 0.000 | < 2e-16 |
| 12 | 1 | 1229.7 | 20.578 | 5.0176 | 98.52 | 0.000 | < 2e-16 |
| 13 | 1 | 1348.5 | 22.467 | 5.5023 | 108.04 | 0.000 | < 2e-16 |
| 14 | 1 | 940.4 | 15.910 | 3.8371 | 75.34 | 0.000 | < 2e-16 |
| 15-2 | 1 | 214.3 | 3.730 | 0.8743 | 17.17 | 0.000 | 3.72e-05 |
| Total |  |  |  | 49.73 |  |  |  |

80 μM haloperidol:

|  | df | Type III SS | LOD | %var | F value | Pvalue(Chi2) | Pvalue(F) |
| --- | --- | --- | --- | --- | --- | --- | --- |
| 1L | 1 | 2382.3 | 31.013 | 6.9971 | 152.033 | 0.000 | < 2e-16 |
| 1R | 1 | 9059.9 | 99.784 | 26.6095 | 578.175 | 0.000 | < 2e-16 |
| 2 | 1 | 425.5 | 5.876 | 1.2497 | 27.154 | 0.000 | 2.29e-07 |
| 7 | 1 | 900.0 | 12.245 | 2.6433 | 57.434 | 0.000 | 8.05e-14 |
| 12 | 1 | 281.5 | 3.904 | 0.8267 | 17.962 | 0.000 | 2.47e-05 |
| 13 | 1 | 1433.1 | 19.183 | 4.2090 | 91.453 | 0.000 | < 2e-16 |
| 14 | 1 | 3161.0 | 40.255 | 9.2841 | 201.726 | 0.000 | < 2e-16 |
| 15-2 | 1 | 116.8 | 1.629 | 0.3431 | 7.455 | 0.006 | 0.00644 |
| 15-1 | 1 | 357.9 | 4.952 | 1.0511 | 22.838 | 0.000 | 2.03e-06 |
| Total |  |  |  | 54.67 |  |  |  |

120 μM haloperidol:

|  | df | Type III SS | LOD | %var | F value | Pvalue(Chi2) | Pvalue(F) |
| --- | --- | --- | --- | --- | --- | --- | --- |
| 1L | 1 | 4662 | 35.57 | 9.023 | 176.97 | 0.000 | < 2e-16 |
| 1R | 1 | 11051 | 76.42 | 21.390 | 419.56 | 0.000 | < 2e-16 |
| 2 | 1 | 1509 | 12.17 | 2.922 | 57.31 | 0.000 | 8.53e-14 |
| 14 | 1 | 4135 | 31.83 | 8.003 | 156.97 | 0.000 | < 2e-16 |
| 15-1 | 1 | 4346 | 33.34 | 8.412 | 165.01 | 0.000 | < 2e-16 |
| Total |  |  |  | 49.53 |  |  |  |

160 μM haloperidol:

|  | df | Type III SS | LOD | %var | F value | Pvalue(Chi2) | Pvalue(F) |
| --- | --- | --- | --- | --- | --- | --- | --- |
| 1L | 1 | 2037.2 | 17.303 | 5.3601 | 82.38 | 0.000 | < 2e-16 |
| 1R | 1 | 4409.1 | 35.841 | 11.6005 | 178.30 | 0.000 | < 2e-16 |
| 2 | 1 | 500.1 | 4.378 | 1.3158 | 20.22 | 0.000 | 7.71e-06 |
| 13 | 1 | 262.9 | 2.313 | 0.6918 | 10.63 | 0.001 | 0.00115 |
| 14 | 1 | 3403.9 | 28.178 | 8.9557 | 137.65 | 0.000 | < 2e-16 |
| 15-1 | 1 | 3126.3 | 26.014 | 8.2255 | 126.43 | 0.000 | < 2e-16 |
| Total |  |  |  | 35.85 |  |  |  |

200 μM haloperidol:

|  | df | Type III SS | LOD | %var | F value | Pvalue(Chi2) | Pvalue(F) |
| --- | --- | --- | --- | --- | --- | --- | --- |
| 1L | 1 | 155.0 | 5.754 | 2.387 | 26.72 | 0.000 | 2.85e-07 |
| 1R | 1 | 179.9 | 6.664 | 2.770 | 31.01 | 0.000 | 3.31e-08 |
| 14 | 1 | 291.9 | 10.711 | 4.495 | 50.32 | 0.000 | 2.49e-12 |
| 15-1 | 1 | 159.2 | 5.907 | 2.451 | 27.44 | 0.000 | 1.98e-07 |
| Total |  |  |  | 12.01 |  |  |  |
