# Supplementary material for: Genetic Basis of Haloperidol Resistance in Saccharomyces cerevisiae Is Complex and Dose Dependent
Source: PLoS Genet. 2014 Dec 18;10(12):e1004894. doi: 10.1371/journal.pgen.1004894 (PMC4270474; doi:10.1371/journal.pgen.1004894)
Supplement: S2 Table — Polymorphisms in the coding region of gene SWH1 (YAR042W) between BY and RM. (DOCX) [file pgen.1004894.s003.docx]

**Table S2. Polymorphisms in the coding region of gene *SWH1* (*YAR042W*) between BY and RM.**

| **Genomic position** | **Amino Acid position** | **SNP type** | **Amino Acid change (BY/RM)** | **Codon change (BY/RM)** |
| --- | --- | --- | --- | --- |
| 192687 | 25 | SYNONYMOUS | R/R | CGC/CGT |
| 192789 | 59 | SYNONYMOUS | A/A | GCC/GCT |
| 192948 | 112 | SYNONYMOUS | I/I | ATC/ATA |
| 193254 | 214 | SYNONYMOUS | R/R | CGT/CGC |
| 193446 | 278 | SYNONYMOUS | L/L | TTG/TTA |
| 193494 | 294 | SYNONYMOUS | F/F | TTC/TTT |
| 193625 | 338 | NON-SYNONYMOUS | D/V | GAT/GTT |
| 194095 | 495 | NON-SYNONYMOUS | T/A | ACT/GCT |
| 194098 | 496 | NON-SYNONYMOUS | P/S | CCC/TCC |
| 194160 | 516 | SYNONYMOUS | D/D | GAT/GAC |
| 194163 | 517 | SYNONYMOUS | D/D | GAT/GAC |
| 194235 | 541 | SYNONYMOUS | N/N | AAC/AAT |
| 194284 | 558 | NON-SYNONYMOUS | L/I | CTA/ATA |
| 194469 | 619 | SYNONYMOUS | T/T | ACC/ACT |
| 194493 | 627 | SYNONYMOUS | V/V | GTT/GTA |
| 194620 | 670 | NON-SYNONYMOUS | V/I | GTT/ATT |
| 194694 | 694 | SYNONYMOUS | T/T | ACC/ACT |
| 194861 | 750 | NON-SYNONYMOUS | E/V | GAA/GTA |
| 195453 | 947 | SYNONYMOUS | P/P | CCT/CCC |
| 195671 | 1020 | NON-SYNONYMOUS | D/G | GAT/GGT |
| 195866 | 1085 | NON-SYNONYMOUS | S/L | TCG/TTG |
| 195904 | 1098 | NON-SYNONYMOUS | I/V | ATA/GTA |

6bp insertion in SWH1 (two alternative alignments between BY and RM alleles):

YAR042W AATGACGATGATGATTATGATGAT------GATGATGAAAGTAGACCCCT 1430

SWH1-RM AATAATAATAATGACGATGATGATTATGATGATGATGAAAGTAGACCCCT 1436

*** * ** **** ******** ********************

YAR042W GATAATAATAATAATAATAATAATG------ACGATGATGATTATGATGA 1409

SWH1-RM GATAATAATAATAATAATAATAATAATAATAATGACGATGATGATTATGA 1415

************************ * ** ****** ** ****
