# Supplementary material for: Genetic Basis of Haloperidol Resistance in Saccharomyces cerevisiae Is Complex and Dose Dependent
Source: PLoS Genet. 2014 Dec 18;10(12):e1004894. doi: 10.1371/journal.pgen.1004894 (PMC4270474; doi:10.1371/journal.pgen.1004894)
Supplement: S3 Table — QTL models incorporating significant two-way QTL interactions (drop one QTL at a time ANOVA tables). (DOCX) [file pgen.1004894.s004.docx]

**Table S3. QTL models incorporating significant two-way QTL interactions (drop one QTL at a time ANOVA tables).**

40 μM haloperidol:

|  | df | Type III SS | LOD | %var | F value | Pvalue(Chi2) | Pvalue(F) |
| --- | --- | --- | --- | --- | --- | --- | --- |
| 1L | 3 | 1613.12 | 33.523 | 6.5821 | 54.656 | 0.000 | < 2e-16 |
| 1R | 7 | 7310.43 | 122.240 | 29.8290 | 106.154 | 0.000 | < 2e-16 |
| 2 | 2 | 274.59 | 6.086 | 1.1204 | 13.955 | 0.000 | 1.06e-06 |
| 7 | 2 | 1530.09 | 31.919 | 6.2433 | 77.764 | 0.000 | < 2e-16 |
| 12 | 3 | 1588.41 | 33.047 | 6.4813 | 53.819 | 0.000 | < 2e-16 |
| 13 | 5 | 2856.12 | 56.218 | 11.6539 | 58.063 | 0.000 | < 2e-16 |
| 14 | 3 | 1827.74 | 37.616 | 7.4578 | 61.928 | 0.000 | < 2e-16 |
| 15-2 | 1 | 134.48 | 3.002 | 0.5487 | 13.670 | 0.000 | 0.00023 |
| 1L:1R | 1 | 246.08 | 5.462 | 1.0041 | 25.013 | 0.000 | 6.75e-07 |
| 1L:13 | 1 | 170.53 | 3.800 | 0.6958 | 17.334 | 0.000 | 3.41e-05 |
| 1R:2 | 1 | 77.48 | 1.735 | 0.3161 | 7.875 | 0.005 | 0.00511 |
| 1R:7 | 1 | 226.26 | 5.027 | 0.9232 | 22.998 | 0.000 | 1.87e-06 |
| 1R:12 | 1 | 167.70 | 3.737 | 0.6843 | 17.046 | 0.000 | 3.96e-05 |
| 1R:13 | 1 | 738.15 | 15.987 | 3.0119 | 75.030 | 0.000 | < 2e-16 |
| 1R:14 | 1 | 379.02 | 8.356 | 1.5465 | 38.526 | 0.000 | 7.97e-10 |
| 12:13 | 1 | 155.17 | 3.460 | 0.6331 | 15.772 | 0.000 | 7.67e-05 |
| 13:14 | 1 | 387.34 | 8.536 | 1.5805 | 39.372 | 0.000 | 5.26e-10 |

80 μM haloperidol:

|  | df | Type III SS | LOD | %var | F value | Pvalue(Chi2) | Pvalue(F) |
| --- | --- | --- | --- | --- | --- | --- | --- |
| 1L | 3 | 2947.75 | 43.627 | 8.6577 | 72.719 | 0.000 | < 2e-16 |
| 1R | 6 | 10197.24 | 123.858 | 29.9499 | 125.780 | 0.000 | < 2e-16 |
| 2 | 2 | 519.77 | 8.360 | 1.5266 | 19.233 | 0.000 | 6.42e-09 |
| 7 | 1 | 960.20 | 15.200 | 2.8202 | 71.062 | 0.000 | < 2e-16 |
| 12 | 4 | 690.98 | 11.045 | 2.0295 | 12.785 | 0.000 | 3.80e-10 |
| 13 | 5 | 2663.33 | 39.782 | 7.8224 | 39.422 | 0.000 | < 2e-16 |
| 14 | 4 | 4296.54 | 60.987 | 12.6192 | 79.495 | 0.000 | < 2e-16 |
| 15-2 | 2 | 138.29 | 2.256 | 0.4062 | 5.117 | 0.006 | 0.00615 |
| 15-1 | 2 | 459.62 | 7.409 | 1.3499 | 17.008 | 0.000 | 5.49e-08 |
| 1L:1R | 1 | 153.83 | 2.508 | 0.4518 | 11.384 | 0.001 | 0.00077 |
| 1L:15-1 | 1 | 120.57 | 1.968 | 0.3541 | 8.923 | 0.003 | 0.00289 |
| 1R:2 | 1 | 104.53 | 1.708 | 0.3070 | 7.736 | 0.005 | 0.00552 |
| 1R:12 | 1 | 108.45 | 1.771 | 0.3185 | 8.026 | 0.004 | 0.00471 |
| 1R:13 | 1 | 384.76 | 6.220 | 1.1301 | 28.476 | 0.000 | 1.18e-07 |
| 1R:14 | 1 | 403.01 | 6.510 | 1.1837 | 29.826 | 0.000 | 6.00e-08 |
| 12:13 | 1 | 74.55 | 1.219 | 0.2190 | 5.517 | 0.018 | 0.01903 |
| 12:14 | 1 | 211.93 | 3.448 | 0.6225 | 15.685 | 0.000 | 8.03e-05 |
| 13:14 | 1 | 504.38 | 8.118 | 1.4814 | 37.328 | 0.000 | 1.44e-09 |
| 13:15-2 | 1 | 92.93 | 1.519 | 0.2729 | 6.877 | 0.008 | 0.00887 |

120 μM haloperidol:

|  | df | Type III SS | LOD | %var | F value | Pvalue(Chi2) | Pvalue(F) |
| --- | --- | --- | --- | --- | --- | --- | --- |
| 1L | 1 | 4884.6 | 38.351 | 9.4543 | 190.911 | 0.000 | < 2e-16 |
| 1R | 2 | 11060.5 | 78.762 | 21.4077 | 216.144 | 0.000 | < 2e-16 |
| 2 | 1 | 1500.6 | 12.521 | 2.9044 | 58.649 | 0.000 | 4.5e-14 |
| 14 | 2 | 4527.2 | 35.764 | 8.7625 | 88.471 | 0.000 | < 2e-16 |
| 15-1 | 1 | 4198.7 | 33.358 | 8.1266 | 164.101 | 0.000 | < 2e-16 |
| 13 | 3 | 527.1 | 4.482 | 1.0203 | 6.867 | 0.000 | 0.000140 |
| 12 | 2 | 346.8 | 2.959 | 0.6713 | 6.777 | 0.001 | 0.001193 |
| 15-2 | 2 | 231.8 | 1.982 | 0.4486 | 4.529 | 0.010 | 0.011016 |
| 1R:13 | 1 | 294.2 | 2.512 | 0.5694 | 11.497 | 0.001 | 0.000725 |
| 14:12 | 1 | 340.8 | 2.908 | 0.6595 | 13.318 | 0.000 | 0.000277 |
| 13:15-2 | 1 | 185.5 | 1.588 | 0.3590 | 7.250 | 0.007 | 0.007210 |

160 μM haloperidol:

|  | df | Type III SS | LOD | %var | F value | Pvalue(Chi2) | Pvalue(F) |
| --- | --- | --- | --- | --- | --- | --- | --- |
| 1L | 3 | 2998.0 | 28.269 | 7.8878 | 45.81 | 0.000 | < 2e-16 |
| 1R | 4 | 6109.0 | 54.146 | 16.0729 | 70.01 | 0.000 | < 2e-16 |
| 2 | 1 | 583.1 | 5.797 | 1.5342 | 26.73 | 0.000 | 2.84e-07 |
| 13 | 1 | 307.3 | 3.074 | 0.8084 | 14.09 | 0.000 | 0.000185 |
| 14 | 4 | 5049.4 | 45.677 | 13.2851 | 57.86 | 0.000 | < 2e-16 |
| 15-1 | 3 | 4397.5 | 40.296 | 11.5700 | 67.19 | 0.000 | < 2e-16 |
| 1L:1R | 1 | 301.8 | 3.020 | 0.7942 | 13.84 | 0.000 | 0.000211 |
| 1L:14 | 1 | 674.5 | 6.691 | 1.7747 | 30.92 | 0.000 | 3.47e-08 |
| 1R:14 | 1 | 715.8 | 7.094 | 1.8832 | 32.81 | 0.000 | 1.35e-08 |
| 1R:15-1 | 1 | 813.1 | 8.041 | 2.1393 | 37.27 | 0.000 | 1.48e-09 |
| 14:15-1 | 1 | 470.9 | 4.693 | 1.2389 | 21.58 | 0.000 | 3.85e-06 |

200 μM haloperidol:

|  | df | Type III SS | LOD | %var | F value | Pvalue(Chi2) | Pvalue(F) |
| --- | --- | --- | --- | --- | --- | --- | --- |
| 1L | 3 | 365.02 | 14.462 | 5.6202 | 22.731 | 0.000 | 3.13e-14 |
| 1R | 3 | 315.72 | 12.564 | 4.8610 | 19.661 | 0.000 | 2.21e-12 |
| 14 | 4 | 642.81 | 24.850 | 9.8972 | 30.023 | 0.000 | < 2e-16 |
| 15-1 | 4 | 390.54 | 15.437 | 6.0131 | 18.240 | 0.000 | 1.82e-14 |
| 1L:14 | 1 | 119.62 | 4.847 | 1.8417 | 22.347 | 0.000 | 2.61e-06 |
| 1L:15-1 | 1 | 72.17 | 2.938 | 1.1112 | 13.483 | 0.000 | 0.000254 |
| 1R:14 | 1 | 103.27 | 4.191 | 1.5901 | 19.294 | 0.000 | 1.24e-05 |
| 1R:15-1 | 1 | 41.63 | 1.699 | 0.6409 | 7.777 | 0.005 | 0.005394 |
| 14:15-1 | 1 | 122.35 | 4.957 | 1.8839 | 22.858 | 0.000 | 2.01e-06 |
