# Supplementary material for: Genetic Basis of Haloperidol Resistance in Saccharomyces cerevisiae Is Complex and Dose Dependent
Source: PLoS Genet. 2014 Dec 18;10(12):e1004894. doi: 10.1371/journal.pgen.1004894 (PMC4270474; doi:10.1371/journal.pgen.1004894)
Supplement: S4 Table — Effects of SWH1, MKT1, IRA2 genes, the genetic background (BG) and their interactions on growth in haloperidol (200 µM). (DOCX) [file pgen.1004894.s005.docx]

**Table S4. Effects of *SWH1, MKT1, IRA2* genes, the genetic background (BG) and their interactions on growth in haloperidol (200μM).**

| **Coefficients** | **Estimate** | **Std. Error** | **t value** | **Pr(>\|t\|)** |
| --- | --- | --- | --- | --- |
| (Intercept) | 0.83 | 0.02 | 34.35 | < 2e-16 |
| BG(RM) | -0.43 | 0.03 | -12.49 | < 2e-16 |
| *MKT1*(BY) | -0.03 | 0.03 | -0.79 | 0.42962 |
| *IRA2*(RM) | 0.10 | 0.03 | 2.80 | 0.00532 |
| *SWH1*(RM) | 0.07 | 0.03 | 2.14 | 0.03246 |
| BG(RM) : *MKT1*(BY) | -0.09 | 0.05 | -1.95 | 0.05163 |
| BG(RM) : *IRA2*(RM) | -0.42 | 0.05 | -8.63 | < 2e-16 |
| *MKT1*(BY) : *IRA2*(RM) | 0.04 | 0.05 | 0.74 | 0.45923 |
| BG(RM) : *SWH1*(RM) | -0.35 | 0.05 | -7.30 | 9.82e-13 |
| *MKT1*(BY) : *SWH1*(RM) | 0.01 | 0.05 | 0.13 | 0.89712 |
| *IRA2*(RM) : *SWH1*(RM) | -0.01 | 0.05 | -0.17 | 0.86191 |
| BG(RM) : *MKT1*(BY) : *IRA2*(RM) | 0.21 | 0.07 | 3.10 | 0.00204 |
| BG(RM) : *MKT1*(BY) : *SWH1*(RM) | 0.77 | 0.07 | 11.22 | < 2e-16 |
| BG(RM) : *IRA2*(RM) : *SWH1*(RM) | 0.28 | 0.07 | 4.07 | 5.36e-05 |
| *MKT1*(BY) : *IRA2*(RM) : *SWH1*(RM) | 0.01 | 0.07 | 0.12 | 0.90741 |
| BG(RM) : *MKT1*(BY) : *IRA2*(RM) : *SWH1*(RM) | 0.07 | 0.10 | 0.70 | 0.48318 |
