# Supplementary material for: Genetic Basis of Haloperidol Resistance in Saccharomyces cerevisiae Is Complex and Dose Dependent
Source: PLoS Genet. 2014 Dec 18;10(12):e1004894. doi: 10.1371/journal.pgen.1004894 (PMC4270474; doi:10.1371/journal.pgen.1004894)
Supplement: S5 Table — Effects of SWH1, MKT1, IRA2 genes and their interactions on growth in haloperidol (BY background, 200 µM). (DOCX) [file pgen.1004894.s006.docx]

**Table S5. Effects of *SWH1, MKT1, IRA2* genes and their interactions on growth in haloperidol (BY background, 200μM).**

| **Coefficients** | **Estimate** | **Std. Error** | **t value** | **Pr(>\|t\|)** |
| --- | --- | --- | --- | --- |
| (Intercept) | 0.828446 | 0.016667 | 49.705 | < 2e-16 |
| *MKT1*(BY) | -0.027353 | 0.023915 | -1.144 | 0.25371 |
| *IRA2*(RM) | 0.095430 | 0.023571 | 4.049 | 6.69e-05 |
| *SWH1*(RM) | 0.073130 | 0.023571 | 3.103 | 0.00212 |
| *MKT1*(BY) : *IRA2*(RM) | 0.035987 | 0.033579 | 1.072 | 0.28478 |
| *MKT1*(BY) : *SWH1*(RM) | 0.006286 | 0.033579 | 0.187 | 0.85164 |
| *IRA2*(RM) : *SWH1*(RM) | -0.008394 | 0.033335 | -0.252 | 0.80136 |
| *MKT1*(BY) : *IRA2*(RM) : *SWH1*(RM) | 0.007967 | 0.047315 | 0.168 | 0.86641 |
