# Supplementary material for: Genetic Basis of Haloperidol Resistance in Saccharomyces cerevisiae Is Complex and Dose Dependent
Source: PLoS Genet. 2014 Dec 18;10(12):e1004894. doi: 10.1371/journal.pgen.1004894 (PMC4270474; doi:10.1371/journal.pgen.1004894)
Supplement: S6 Table — Tukey-HSD test of SWH1, MKT1, and IRA2 allelic effects, and their two-way interactions (200 µM haloperidol). (DOCX) [file pgen.1004894.s007.docx]

**Table S6. Tukey-HSD test of *SWH1*, *MKT1*, and *IRA2* allelic effects, and their two-way interactions (200 μM haloperidol).**

Tukey multiple comparisons of means in BY (95% family-wise confidence level)

$mkt1

|  | diff | lwr | upr | p adj |
| --- | --- | --- | --- | --- |
| B-R | -0.005684495 | -0.02881624 | 0.01744725 | 0.6289439 |

$ira2

|  | diff | lwr | upr | p adj |
| --- | --- | --- | --- | --- |
| R-B | 0.1134711 | 0.09033935 | 0.1366028 | 0 |

$swh1

|  | diff | lwr | upr | p adj |
| --- | --- | --- | --- | --- |
| R-B | 0.07686335 | 0.0537316 | 0.0999951 | 0 |

$mkt1:ira2

|  | diff | lwr | upr | p adj |
| --- | --- | --- | --- | --- |
| B:B-R:B | -0.02959792 | -0.07270353 | 0.01350770 | 0.2877350 |
| R:R-R:B | 0.09068799 | 0.04788701 | 0.13348897 | 0.0000006 |
| B:R-R:B | 0.10699310 | 0.06419212 | 0.14979408 | 0.0000000 |
| R:R-B:B | 0.12028591 | 0.07718029 | 0.16339152 | 0.0000000 |
| B:R-B:B | 0.13659102 | 0.09348541 | 0.17969664 | 0.0000000 |
| B:R-R:R | 0.01630511 | -0.02649586 | 0.05910609 | 0.7583507 |

$mkt1:swh1

|  | diff | lwr | upr | p adj |
| --- | --- | --- | --- | --- |
| B:B-R:B | -0.014439750 | -0.05754537 | 0.02866586 | 0.8224888 |
| R:R-R:B | 0.068768918 | 0.02596794 | 0.11156990 | 0.0002545 |
| B:R-R:B | 0.070641354 | 0.02784038 | 0.11344233 | 0.0001597 |
| R:R-B:B | 0.083208668 | 0.04010305 | 0.12631428 | 0.0000064 |
| B:R-B:B | 0.085081104 | 0.04197549 | 0.12818672 | 0.0000037 |
| B:R-R:R | 0.001872436 | -0.04092854 | 0.04467341 | 0.9994819 |

$ira2:swh1

|  | diff | lwr | upr | p adj |
| --- | --- | --- | --- | --- |
| R:B-B:B | 0.11930906 | 0.07620344 | 0.162414670 | 0.0000000 |
| B:R-B:B | 0.08215817 | 0.03905256 | 0.125263789 | 0.0000085 |
| R:R-B:B | 0.19094848 | 0.14784286 | 0.234054093 | 0.0000000 |
| B:R-R:B | -0.03715088 | -0.07995186 | 0.005650097 | 0.1142386 |
| R:R-R:B | 0.07163942 | 0.02883845 | 0.114440401 | 0.0001240 |
| R:R-B:R | 0.10879030 | 0.06598933 | 0.151591281 | 0.0000000 |

Tukey multiple comparisons of means in RM (95% family-wise confidence level)

$mkt1

|  | diff | lwr | upr | p adj |
| --- | --- | --- | --- | --- |
| B-R | 0.4080682 | 0.3667113 | 0.449425 | 0 |

$ira2

|  | diff | lwr | upr | p adj |
| --- | --- | --- | --- | --- |
| R-B | -0.04315263 | -0.08450946 | -0.001795791 | 0.040909 |

$swh1

|  | diff | lwr | upr | p adj |
| --- | --- | --- | --- | --- |
| R-B | 0.2616535 | 0.2202966 | 0.3030103 | 0 |

$mkt1:ira2

|  | diff | lwr | upr | p adj |
| --- | --- | --- | --- | --- |
| B:B-R:B | 0.26502838 | 0.1882372 | 0.3418195 | 0.0000000 |
| R:R-R:B | -0.18619240 | -0.2629835 | -0.1094013 | 0.0000000 |
| B:R-R:B | 0.36491553 | 0.2881244 | 0.4417067 | 0.0000000 |
| R:R-B:B | -0.45122078 | -0.5280119 | -0.3744296 | 0.0000000 |
| B:R-B:B | 0.09988715 | 0.0230960 | 0.1766783 | 0.0048627 |
| B:R-R:R | 0.55110793 | 0.4743168 | 0.6278991 | 0.0000000 |

$mkt1:swh1

|  | diff | lwr | upr | p adj |
| --- | --- | --- | --- | --- |
| B:B-R:B | 0.00197213 | -0.07481901 | 0.07876327 | 0.9998949 |
| R:R-R:B | -0.14444254 | -0.22123368 | -0.06765139 | 0.0000115 |
| B:R-R:B | 0.66972163 | 0.59293049 | 0.74651278 | 0.0000000 |
| R:R-B:B | -0.14641467 | -0.22320581 | -0.06962352 | 0.0000085 |
| B:R-B:B | 0.66774950 | 0.59095836 | 0.74454065 | 0.0000000 |
| B:R-R:R | 0.81416417 | 0.73737303 | 0.89095532 | 0.0000000 |

$ira2:swh1

|  | diff | lwr | upr | p adj |
| --- | --- | --- | --- | --- |
| R:B-B:B | -0.1967580 | -0.27354911 | -0.1199668 | 0.0000000 |
| B:R-B:B | 0.1080481 | 0.03125699 | 0.1848393 | 0.0018579 |
| R:R-B:B | 0.2185009 | 0.14170971 | 0.2952920 | 0.0000000 |
| B:R-R:B | 0.3048061 | 0.22801496 | 0.3815973 | 0.0000000 |
| R:R-R:B | 0.4152588 | 0.33846768 | 0.4920500 | 0.0000000 |
| R:R-B:R | 0.1104527 | 0.03366157 | 0.1872439 | 0.0013811 |
